# Stem cell transplantation for hemoglobinopathy Vila Real

**DOI:** 10.1007/s00277-020-04237-6

**Published:** 2020-09-03

**Authors:** Justin Hasenkamp, Joachim Riggert, Wolfram Jung, Hristo Boyadzhiev, Jakob Valk, Lorenz Trümper, Gerald Wulf

**Affiliations:** 1 Hematology and Medical Oncology, University Medicine Goettingen, Robert-Koch-Str. 40, D–37099 Goettingen, Germany; 2 Department of Transfusion Medicine, University Medicine Goettingen, D–37099 Goettingen, Germany

Dear Editor,

Hemoglobinopathy Vila Real (HbVR) is a rare congenital disease caused by a hemoglobin beta chain P36H mutation [1, 2]. The hemoglobin variant is associated with increased oxygen affinity, resulting in reduced oxygen delivery to target tissues, painful hypoxic organ damage, and compensatory erythrocytosis. Diagnosis is based on clinical presentation of erythrocytosis with high oxygen saturation and tissue hypoxia, to be confirmed by hemoglobin sequence analysis. Hitherto, therapy of symptomatic patients consists of phlebotomy plus transfusions to alleviate symptoms.

We report the case of a 47-year-male diagnosed with HbVR 10 years before in the work-up of erythrocytosis. When the patient experienced progressive pain attacks in the head, testis, and muscles, phlebotomy and transfusions of cumulative 168 erythrocyte concentrates were started, without amelioration in the recent year. As yet, antibodies against red blood cells were not detected to this point of time.

Bone marrow transplantation (BMT) was offered to the patient to avoid the long-term complications of transfusion dependency and disease-related end-organ damage [3]. To avoid disease-related ischemic injury and reperfusion events during transplantation, similar to symptoms of sickle cell crisis, but with different pathomechanism, complete blood exchanges were performed before the preparative regimen was started. Following conditioning with thiotepa, fludarabine, and treosulfan, bone marrow from a HLA-matched unrelated donor containing 1.5 × 10^8^ leukocytes/kg body weight (BW) was transfused after depletion of erythrocytes and plasma. Graft-versus-host-disease prophylaxis consisted of anti-thymocyte-globulin, cyclosporin A (CSA), and mycophenolate mofetil. During aplasia, CMV reactivation and a sepsis responded to treatment. Leukocyte engraftment occurred 21 days post-BMT with complete donor chimerism. The patient remained dependent on transfusion for erythrocytes and thrombocytes. Bone marrow investigation on day 27 revealed graft failure suspected to be due to the low cell count in the transplanted product. Fifty-one days post-BMT, we transfused 4.0 × 10^6^ CD34+ cells/kg BW purified from the mobilized peripheral blood stem cells of the original donor. From day 8 after the stem cell boost, the patient was transfusion independent and the reticulocytes rose with continuous complete donor chimerism.

Further complications post-BMT resembled typical sickle cell complications: seizures and visual impairments compatible with posterior reversible encephalopathy syndrome (PRES) by NMR imaging (Fig. 1), diabetes insipidus centralis due to the PRES or CSA, and diabetes insipidus renalis due to foscarnet turned out to be manageable. Following recovery, further follow-up was uneventful through 2.5 years, without immunosuppression and according to hemoglobin analysis complete correction of the hemoglobinopathy.

**Fig. 1:**
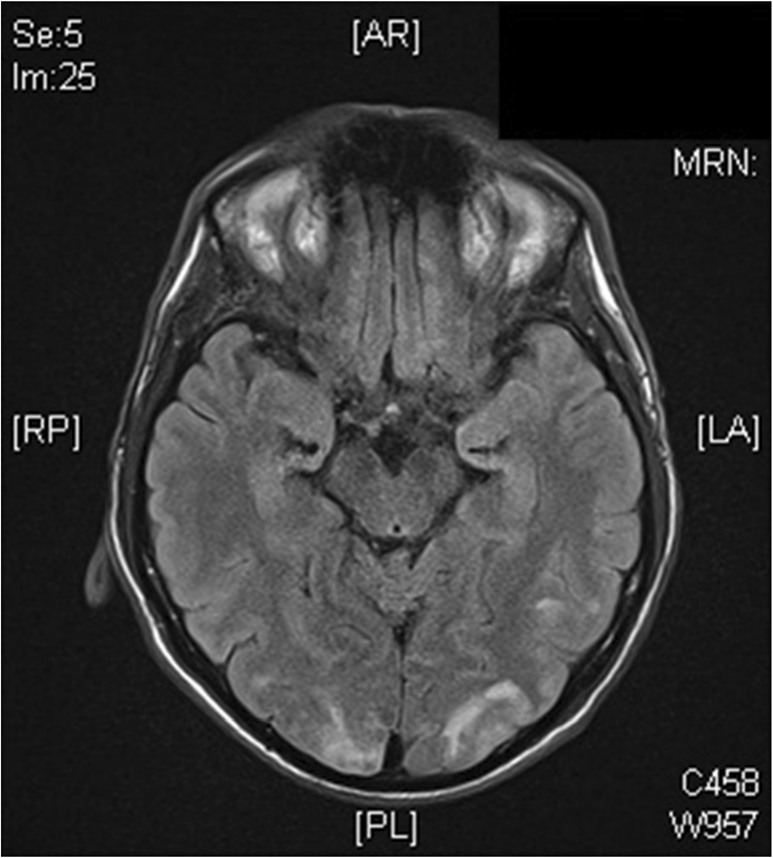
NRM brain image of the patient 40 days post-BMT with occipital edema compatible with posterior reversible encephalopathy syndrome (PRES)

This is the first report of allogeneic hematopoietic stem cell transplantation (HSCT) for a patient with HbVR, a condition associated with severely reduced quality of life and high prevalence of end-organ complications. The curative potential of HSCT in hemoglobinopathies is well-established [3] but is hampered by the dilemma to outweigh the benefits from definite cure versus the transplant-related risks for. Particular in adult patients with advanced disease, comorbidities precipitate complications [4]. PRES is elsewise common in pediatric hemoglobinopathy patients receiving HSCT [5–7].

In summary, the rarity and clinical heterogeneity of HbVR will always demand an individual approach. For severely affected patients, we report HSCT as a successful option.

## Data Availability

“Not applicable” for that section.
